# Non-linear Methods Predominant in Fetal Heart Rate Analysis: A Systematic Review

**DOI:** 10.3389/fmed.2021.661226

**Published:** 2021-11-30

**Authors:** Maria Ribeiro, João Monteiro-Santos, Luísa Castro, Luís Antunes, Cristina Costa-Santos, Andreia Teixeira, Teresa S. Henriques

**Affiliations:** ^1^Institute for Systems and Computer Engineering, Technology and Science, Porto, Portugal; ^2^Computer Science Department, Faculty of Sciences, University of Porto, Porto, Portugal; ^3^Centre for Health Technology and Services Research, Faculty of Medicine University of Porto, Porto, Portugal; ^4^Department of Community Medicine, Information and Health Decision Sciences, Faculty of Medicine, University of Porto, Porto, Portugal; ^5^School of Health of Polytechnic of Porto, Porto, Portugal; ^6^Instituto Politécnico de Viana do Castelo, Viana do Castelo, Portugal

**Keywords:** fetal heart rate, non-linear methods, entropy, data compression, fractal analysis, wavelet analysis, systematic review

## Abstract

The analysis of fetal heart rate variability has served as a scientific and diagnostic tool to quantify cardiac activity fluctuations, being good indicators of fetal well-being. Many mathematical analyses were proposed to evaluate fetal heart rate variability. We focused on non-linear analysis based on concepts of chaos, fractality, and complexity: entropies, compression, fractal analysis, and wavelets. These methods have been successfully applied in the signal processing phase and increase knowledge about cardiovascular dynamics in healthy and pathological fetuses. This review summarizes those methods and investigates how non-linear measures are related to each paper's research objectives. Of the 388 articles obtained in the PubMed/Medline database and of the 421 articles in the Web of Science database, 270 articles were included in the review after all exclusion criteria were applied. While approximate entropy is the most used method in classification papers, in signal processing, the most used non-linear method was Daubechies wavelets. The top five primary research objectives covered by the selected papers were detection of signal processing, hypoxia, maturation or gestational age, intrauterine growth restriction, and fetal distress. This review shows that non-linear indices can be used to assess numerous prenatal conditions. However, they are not yet applied in clinical practice due to some critical concerns. Some studies show that the combination of several linear and non-linear indices would be ideal for improving the analysis of the fetus's well-being. Future studies should narrow the research question so a meta-analysis could be performed, probing the indices' performance.

## 1. Introduction

Worldwide, it is estimated that the number of fetal deaths after week 20 of gestational age is around 2.6 million per year. Although the numbers have been decreasing in the past decades, the stillbirths' rate still ranges from about 1 in 250 births in developed countries and 1 per 33 in South Asia and Sub-Saharan Africa (data from 2009), according to Cousens et al. (1).

Cardiotocography (CTG) combines fetal heart rate (fHR) measurement, obtained through a uterine contraction monitoring probe and a Doppler ultrasound probe for fHR, recorded using an abdominal pressure transducer. In developed countries, clinical decisions during labor are firmly based on fHR monitoring (2, 3), being CTG the most used tool to assess fetal well-being since the early '60s according to Spencer (4). However, the information provided by CTG is limited since a complete electrocardiogram (ECG) signal of the fetus is not available. Moreover, the CTG is highly sensitive to both fetal and maternal movement. The use of an electrode placed on the fetus's scalp is more reliable as it retrieves fetal electrocardiogram, containing not only fHR but also other crucial clinical parameters (5, 6). On the other hand, this is an invasive method only possible during labor, after the beginning of cervical dilatation and the membranes' rupture, carrying with it risks of infection (7, 8). However, other methods for fetal monitoring are used such as fetal phonocardiography (9–11), fetal echocardiography (12, 13), and fetal magnetocardiography (14, 15). Each one of the methods has its own advantages and disadvantages. For more detail on this matter, see Jaros et al. (16) and Hoyer et al. (17).

Electronic fetal monitoring came with high expectations since it offered continuous monitoring, compared to the intermittent auscultation done until then. However, a meta-analysis of large multicenter studies did not prove any significant improvement. Also, electronic fetal monitoring became the main suspect for the increased rate of cesarean sections (18). These procedures result in a slight increase in poor outcomes in low-risk pregnancies. The cesarean sections also require a longer time to heal than a vaginal birth and present increased risks, including baby breathing problems, amniotic fluid embolism, and postpartum bleeding for the mother (19). Despite the importance of the fetus and mother well-being assessment, low concordance between physicians is still present, even among experienced obstetricians, resulting in a high rate of false-positives (2, 20, 21). In daily practice, fHR is subject to the clinician visual interpretation, even when following the guidelines provided by the International Federation of Obstetrics and Gynaecology (FIGO) (22, 23), which although being associated with high sensitivity but low specificity (24), might leads to a chance of more harmful than beneficial adherence to conventional guidelines (25).

The autonomic nervous system (ANS) is involved in the control of almost every organ system, and the beat-to-beat variation of fHR reflects the influence of the fetus' ANS and its components (sympathetic and parasympathetic) and, therefore, is an indicator of fetal well-being (8). A certain level of unpredictable fetal heart rate variability (fHRV) reflects sufficient capabilities of the organism in search of optimal behavior. Reduced fHRV is linked with limited capabilities and mental disorders (26). The linear modeling approaches quantify sympathetic and parasympathetic control mechanisms and their balance by measuring spectral low and high-frequency components. However, it has been shown that not all information carried by beat-to-beat variability can be explained by these components (27). For this matter, in the past couple of decades, and with the fast development of computation, new signal processing, and pattern recognition methodologies have been developed and applied to many different fields, including the analysis of fHRV using non-linear parameters (28, 29). These approaches can reveal relevant clinical information not exposed by temporal or frequency analysis (30).

Variability and complexity are different terms. While a complex system requires variability, the other way around is not guaranteed. For example, a set of random notes in music can be interpreted as having high complexity for its non-predictability, whereas a set of consecutive notes is highly predictable, and both have high variability. Thus, complexity signals, such as those produced by self-regulatory physiological systems, present temporal or spatial structures over a varied range of scales. Because of their non-linearity and non-stationarity, conventional indicators, such as the mean and the standard deviation, do not fulfill their purpose (31). In the end, complexity is a property of any system that quantifies the amount of structured information.

Chaffin et al. (32), in 1991, were the first to use non-linear analyzes in fHR. The authors applied fractal analysis (correlation dimension) to study 12 normal fetuses' well-being in labor. Later, in 1992, Pincus and Viscarello (33) found statistically significant results using approximate entropy (ApEn) when comparing a group of acidemic fetuses with non-acidemic ones. These results supported the hypothesis that regular fHR patterns are associated with acidemia. Datian and Xuemei (34), in 1996, introduced a new wavelet analysis method used to detect fetal electrocardiogram from the abdominal signal and compared to other methods in practice. Signorini et al. (35), in 2005, applied data compression (Lempel Ziv complexity) for the first time in the fHR analysis to improve the early detection of fetal distress conditions such as intrauterine growth restriction. The same authors, also in 2007 (36), used the Lempel Ziv complexity to successfully discriminate between severe intrauterine growth restriction (IUGR) (premature birth) and non-severe IUGR (term delivery) and normal fetuses. In the subsequent year, using a compressor-based clustering algorithm called normalized compression distance (NCD), Santos et al. (37) managed to clustered abnormal and suspicious tracks, regardless of the monitoring system used. Barquero-Pérez et al. (38) also used NCD for automatic detection of perinatal hypoxia.

The main contribution of this article is to provide a systematic review of articles that apply entropy, compression, fractal, and wavelet analysis to study the dynamics of fHR and analyze the research objectives of these articles. As far as we know, there is no systematic review for this purpose in the literature.

We begin by describing the methodology used, specifying the sources of information, the eligibility criteria, the study selection, data extraction, and quality assessment in section 2. Based on the systematic review results, we describe in detail the most commonly used non-linear methods to assess the dynamics of fetal heart rate and analyze how the study of the complexity of fHR has evolved over the years (section 3). In section 4, we describe the most frequent goals in research. We analyze the evolution of the non-linear methods' applications to these objectives and probe how the research objectives are related to non-linear methods. In section 5, we reflect on some open questions regarding the application of non-linear measurements to fHR dynamics. We finish this paper with the main conclusions in section 6.

## 2. Systematic Review Methods

This systematic review focused on original papers that include non-linear analysis, such as complexity measures, fractal approaches, and wavelets, of human fetal heart rate during ante and intrapartum. The online search was performed on Medline, through PubMed, and the Web of Science databases, searching all the papers published until the 4th of October 2020. The following terms were used as descriptors/Mesh: “non-linear dynamics,” “entropy,” “data compression,” “complexity,” “fractals,” “wavelets,” “fetal heart rate,” “foetal heart rate.” The queries used in each database can be found in Table 1. This study was conducted according to the Preferred Reporting Items for Systematic Reviews and Meta-analyses (PRISMA) statement (39). The review protocol was not registered prospectively.

**Table 1:** Online queries in Pubmed and Web of Science.

**Pubmed (https://www.ncbi.nlm.nih.gov/pubmed)**
(“Nonlinear Dynamics”[Mesh] OR “Nonlinear Dynamics”[Title/Abstract] OR Nonlinear[Title/Abstract] OR “Entropy”[Mesh] OR Entropy[Title/Abstract] OR “Data Compression”[Mesh] OR “Data Compression”[Title/Abstract] OR Compression[Title/Abstract] OR complexity[Title/Abstract] OR “fractals”[MeSH Terms] OR fractals[Title/Abstract] OR “Wavelet Analysis”[Mesh] OR “Wavelet Analyses”[Title/Abstract] OR wavelet[Title/Abstract]) AND (“Heart Rate, Fetal”[Mesh] OR “Fetal Heart Rate”[Title/Abstract] OR “foetal heart rate”[Title/Abstract])
**Web of Science (https://www.webofknowledge.com)**
(TS=(“Nonlinear Dynamics” OR nonlinear OR entropy OR Compression OR complexity OR fractal OR wavelet) OR TI=(“Nonlinear Dynamics” OR nonlinear OR entropy OR Compression OR complexity OR fractal OR wavelet)) AND (TI=(“Heart Rate, Fetal”) OR TI=(“Fetal Heart Rate”) OR TI=(“foetal heart rate”) OR TS=(“fetal heart rate”) OR TS=(“foetal heart rate”))

**Figure 1:**
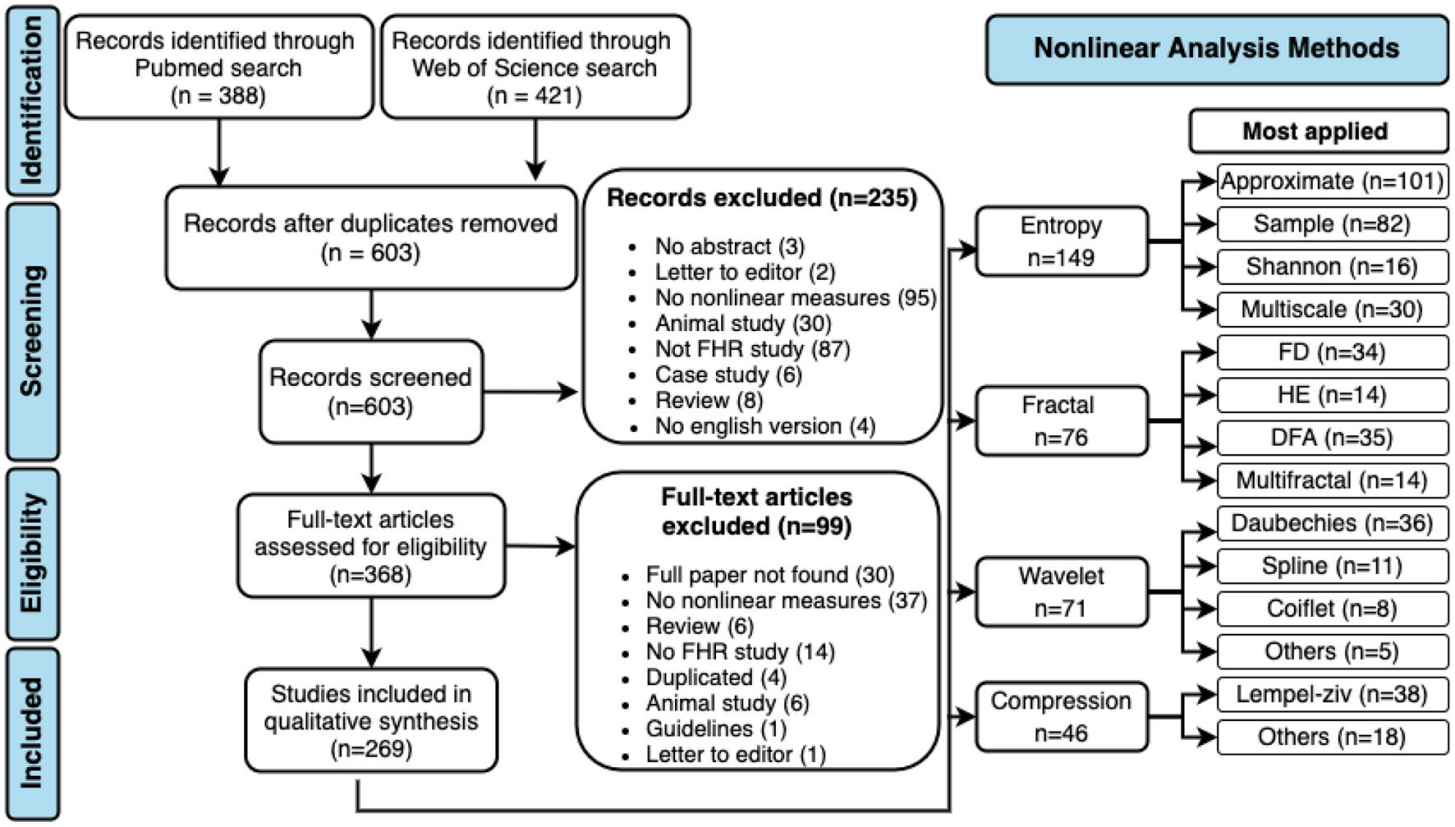
PRISMA flow diagram and non-linear methods most applied in fHR. DFA, detrended fluctuation analysis; FD, fractal dimension; HE, Hurst exponent.

Inclusion criteria for selecting studies were the following: observational or experimental papers presenting complexity analysis of fetal heart rate; abstract found online; reported original research in peer-reviewed journals; at least one measure from the following was used in the analysis (entropy, compression, fractal, or wavelet). Papers using non-human fetal heart rate analysis, papers without an English version, reviews, case studies, dissertations, and thesis were excluded (see Figure 1).

All authors were involved in the selection of studies, data extraction, and quality assessment. Two authors independently assessed each title and abstract found in the databases. The full texts of potentially relevant studies have been retrieved and revised in depth. Disagreements between reviewers were resolved by consensus or by the decision of a third independent reviewer. For each article, the following data were collected: year of publication, study design, objective, sample size, measure(s) used to analyze fHR, and conclusions. Both reviewers made sure that all included papers met the criteria defined in the first stage.

A total of 603 abstracts were assessed, 368 of which retained for full-text screening. Two hundred and seventy papers were then included in the review after meeting all the criteria. Figure 1 contains the PRISMA flow diagram for study selection, including reasons for exclusion. The most used non-linear analysis measures to study the dynamics of fHR obtained in the systematic review are also represented in Figure 1.

## 3. Non-linear Methods

**Figure 2:**
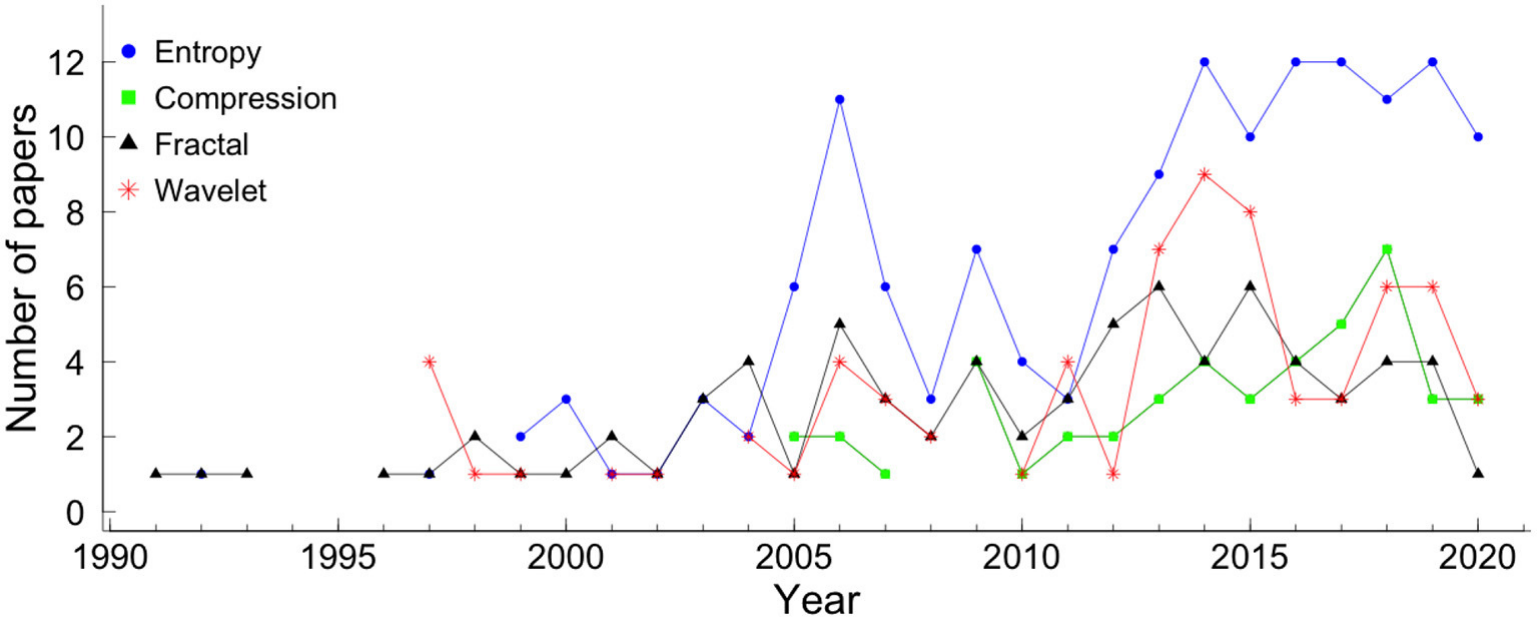
The number of papers, covered by the systematic review, that applied each method. The blue circle represents entropy, the green square represents compression, the black triangle represents fractal analysis, and the red star represents wavelet.

Although linear indices have been extensively used in fetal monitoring for the past decades, it is established that biological systems are more complex than they appear. Non-linear measures based on concepts of chaos, fractality, and complexity have gained space and demonstrated promising results in the analysis of fetal well-being and the prediction of pathologies. The application of non-linear measures to study the dynamics of fHR has increased over the years. The non-linear methods covered by this review are entropy, compression, fractal analysis, and wavelet analysis. The results show that entropy is the most applied measure in fetal heart rate, followed by fractal analysis, wavelet analysis, and the least applied is the compression (see Figures 1, 2). Although the application of entropy methods stands out, we can see that compression and wavelet analysis methods have been increasingly used in recent years (see Figure 2).

In the following sections, the most applied non-linear methods are described. In our systematic review, other non-linear methods were found, such as, Poincaré plot (in 18 papers), symbolic dynamics (in 12 papers), phase rectified signal average (in 10 papers), Lyapunov exponents (in 6 papers), and recurrence plot analysis (in 6 papers). However, due to the reduced number of uses, they were not described in detail. For this review, we decided to describe only the measures most applied to fHR.

### 3.1. Entropy

According to Shannon (40), the information within a signal can be quantified with absolute precision as the amount of unexpected data in the message (defined as entropy). Entropy, a probabilistic complexity measure used to quantify a time series's irregularity, has been widely used in physiological signal analysis. The number of papers that applied each entropy measure per year is shown in Figure 2. The entropy measures that were applied to at least 15 articles were: Shannon entropy (SE), approximate entropy (ApEn), sample entropy (SampEn), and multiscale entropy (MSE).

**Figure 3:**
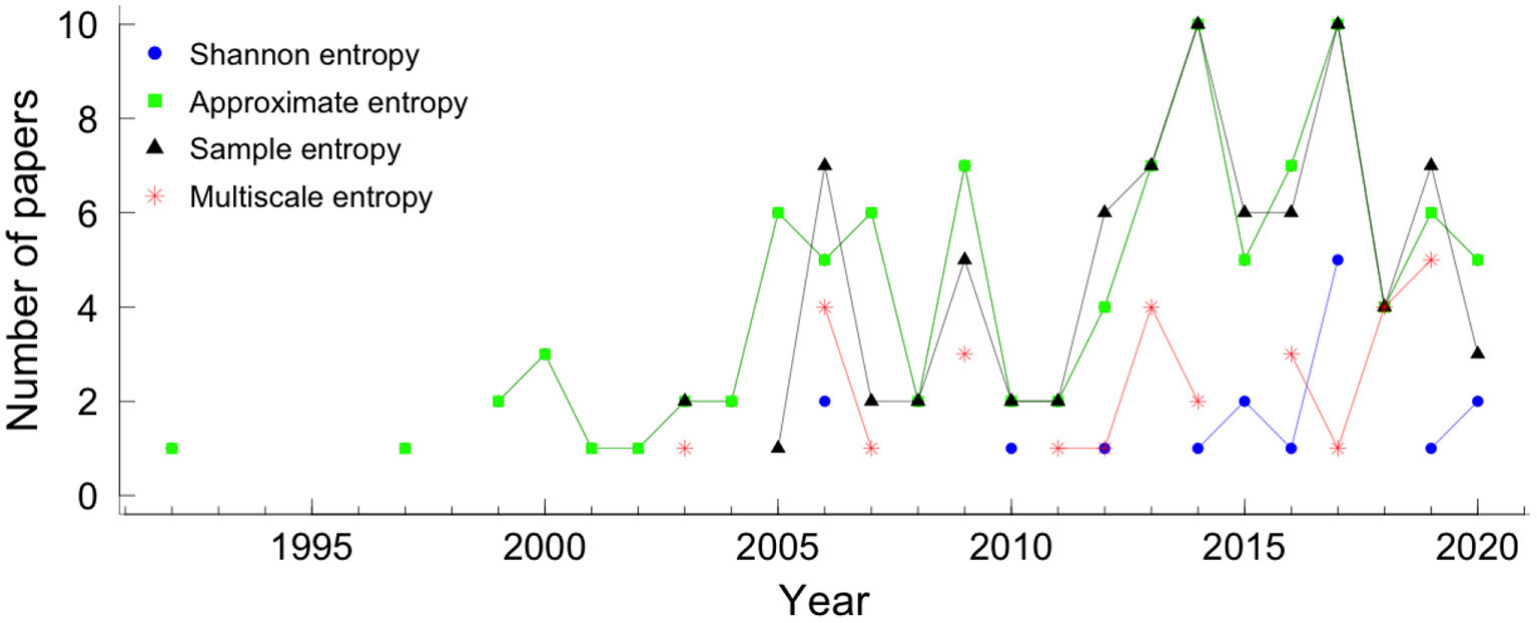
Entropies by year. The colors and symbols represent the different measures of entropy. The blue circle, green square, black triangle, and star red represented Shannon entropy, approximate entropy, sample entropy, and multiscale entropy, respectively.

From all 270 papers included in this review, 149 (55.2%) papers applied entropy: 16 (5.9%) show results with Shannon entropy (SE), 82 (30.4%) used SampEn, 101 (37.4%) used ApEn, and 30 (11.1%) used MSE (see Figure 1). Figure 3 shows the number of papers that applied measures of the entropy by year. ApEn is the most applied measure. However, in recent years the employment of SampEn and ApEn is similar.

In the literature, we found other entropy measures that appeared in less than 15 articles, such as, permutation entropy (41–45), Rnyi entropy (46–48), Kullback-Leibler entropy (41, 42, 49, 50), Kolmogorov-Sinai entropy (30), cross-sample entropy (51, 52), tone-entropy (53), bubble entropy (47), and compression entropy (46).

#### 3.1.1. Shannon Entropy (SE)

In 1948, Shannon (40) proposed the concept of entropy (Shannon entropy - SE) to measure how the information within a signal can be quantified with absolute precision as the amount of unexpected data contained in the message. The Shannon entropy is obtained by:


(1) $$\begin{array}{r} SE=-\underset{i}{\sum }p\left(x\left(i\right)\right)\cdot log\left(p\left(x\left(i\right)\right)\right) \end{array}$$

where *p*(*x*(*i*)) represents the probability of the point *x*(*i*), of a time series *X* = (*x*_1_, *x*_2_, ..., *x*_*N*_).

Though SE was introduced back in 1948, and many new entropies appeared to overcome some of the SE limitations, some authors still applied it in the analysis of fHRV (46, 54).

#### 3.1.2. Approximate Entropy (ApEn)

In 1991, Pincus et al. (55) developed a regularity statistic tool to quantify the amount of regularity and the unpredictability of fluctuations over time-series data. The ApEn is based on the assumption that healthy dynamic stability comes from specific networks' specific mechanisms and properties. When a vulnerable connection arises between systems or within one, it is the disease mechanism, which is characterized by an increase of regularity of the series (56).

Considering a time series *X* = (*x*_1_, *x*_2_, ..., *x*_*N*_), in order to calculate the ApEn the new series of a vector of length *m* (embedding dimension), *X*_*m*_(*i*) = (*x*_*i*_, *x*_*i*+1_, *x*_*i*+2_, …, *x*_*i*+*m*−1_) are constructed for each *i* = 1, …, *N* − *m* + 1. For each vector *X*_*m*_(*i*), the value $C_{m}^{r}\left(i\right)$, where *r* is referred as a tolerance value, is computed as:


(2) $$\begin{array}{r} C_{m}^{r}\left(i\right)=\frac{\text{number of }d\left[X_{i},X_{j}\right]\leq r}{N-m+1},\;\forall j \end{array}$$

Here, the distance between the vector *X*_*m*_(*i*) and its neighbor *X*_*m*_(*j*) is defined as:


(3) $$\begin{array}{r} d\left[X_{m}\left(i\right),X_{m}\left(j\right)\right]=max_{k=1,\ldots ,m}|x\left(i+k-1\right)-x\left(j+k-1\right)| \end{array}$$

Next, the average of the natural logarithm of $C_{m}^{r}\left(i\right)$ is computed for all *i*:


(4) $$\begin{array}{r} \Phi_{m}^{r}=\frac{1}{N-m+1}\overset{N-m+1}{\underset{i=1}{\sum }}ln\left(C_{m}^{r}\left(i\right)\right) \end{array}$$

Since in practice *N* is a finite number, the statistical estimate is computed as:


$$ApEn\left(m,r\right)=\{\begin{array}{ll} \Phi_{m}^{r}-\Phi_{m+1}^{r} & \;\text{for }m>0\\ -\Phi_{1}^{r} & \;\text{for }m=0 \end{array}$$

In the particular case of the ApEn, the most common value is *m* = 2. However, many algorithms were proposed to estimate the smallest sufficient embedding dimension, *m*. One of the most used methods is the “false nearest-neighbors” algorithm proposed by Kennel et al. (57). Though, the limitation of this method relies on the subjective definition of false neighbor (58). To overcome this limitation, Cao (58) proposed a new method.

For estimation of an appropriate time delay various approaches have been proposed. The most used two are the autocorrelation function and the average mutual information function (59). Pincus (60) and Pincus and Goldberger (61) recommends values between 10 and 25% of the standard deviation of the data, hence obtaining a scale-invariant measurement. The approach of choosing a fixed *r* value was also used with success (62, 63). However, the values of entropy in this case are usually highly correlated with the time series standard deviation. Lu et al. (64) showed that ApEn values varied significantly even within the defined range of *r* values and presented a new method for automatic selection of *r* that corresponds to the maximum ApEn value.

#### 3.1.3. Sample Entropy (SampEn)

In 2000, Richman and Moorman (65) proposed the sample entropy (SampEn), with the same purpose as ApEn, to evaluate the randomness of biological time series, in particular, the HR time series. The main limitation of the ApEn is the dependence on the record length, i.e., the ApEn is lower for short records, and if one time series is higher than another, it should not remain higher for all conditions (65). In order to overcome the limitations, the authors proposed a new family of statistics, *SampEn*(*m, r*), which, with some differences, reducing bias specially in short data sets:

1. self-matches are not counted;
2. only the first N-vectors of length are considered;
3. the conditional probabilities are not estimated in a template manner.

To calculate the value of SampEn (65) the parameters *m*, and *r* defined for ApEn are needed. Considering *A* as the number of vector pairs of length *m* + 1 having *d*[*X*_*m*_(*i*), *X*_*m*_(*j*)] ≤ *r*, with *i* ≠ *j* and *B* as the total number of template matches of length *m* also with *i* ≠ *j*, the SampEn is defined by the equation:


(5) $$\begin{array}{r} SampEn=-ln\frac{A}{B} \end{array}$$

This probability measure is computed directly as the logarithm of conditional probability and not from the logarithmic sums ratio, showing relative consistency in cases where ApEn does not (65).

#### 3.1.4. Multiscale Entropy (MSE)

ApEn and SampEn have the disadvantage of outputting a single index concerning the time series's general behavior, thus not revealing its underlying dynamics. MSE has been widely employed in the biomedical signal analysis as it allows measuring signal properties at different time scales (66, 67).

Considering a time series *X* = {*x*_*i*_} of *N* points, it constructs consecutive coarse-grained time series $y_{j}^{\left(\tau \right)}$, replacing τ non-overlapping points by their average. The MSE curve is created by computing the entropy for each of the scales and plotted vs. the scale. The information of the different time scales is clustered in the complexity index defined as the area under the MSE curve.

The estimation of the complexity methodology has to follow the multiscale application requirements, and SampEn was proposed using a tolerance r obtained from the original series and keeping it constant for all scales (67). Other authors were in favor of choosing an individual tolerance level r for each scale (68, 69). For example, the quadratic sample entropy permits a personalized estimation of r for each scale in short data (70).

The physiological interpretation of multiscale complexity is not always clear once, in a complex dynamic system, all scales might be affected by regulating influences (71). Low complexity scales indicate regular patterns with periodicity, but isolated ones would indicate one single frequency oscillation periodicity that usually is not present in complex systems. However, it is typical of the appearance of correlated neighboring scales (41, 67).

### 3.2. Compression

Dynamic systems theory was firstly linked with information theory by Kolmogorov (72), in 1958. Years later, “algorithm information theory” was then independently proposed by three different authors, Solomonoff (73), Kolmogorov (74) and Chaitin (75).

Let *x* be a finite length binary string, U be a universal computer, *l*(*x*) denote the length of the string *x* and U(p) the output of the computer U when presented with a program *p*. The Kolmogorov (or algorithmic) complexity (KC) of a string *x* with respect to a universal computer U, $K_{U}\left(x\right)$, is defined as the shortest description length of *x* over all descriptions interpreted by computer U. In different words, KC quantifies how “random” an individual object is in terms of the number of bits necessary to describe it. For a random string, the output of $K_{U}\left(x\right)$ function will be the original string's length as any compression effort will end in information loss. The more reoccurring patterns, the less complex the signal is. Although this concept is objective, its applicability is limited to the fact that it is not computable. Compressors are a close upper-bounded approximation of the $K_{U}\left(x\right)$ function. For over 30 years, data compression software has been developed for data storage and transmission efficiency purposes, and more recently, compression has been utilized in health research.

The innumerous compressors found in the literature can be divided into two big groups: lossless or lossy. The former group is composed of compressors in which, after being decompressed, all original information is restored. For the lossy group, this is not guaranteed, particularly for redundant information. The most applied compressors in health research belong to the first group.

The Lempel–Ziv algorithm was introduced, in 1976, by Lempel and Ziv (76) based on 'the concept of encoding future segments of the source output via maximum-length copying from a buffer containing the recent past output.' It was the starting point for different compressors such as the Lempel–Ziv–Markov chain algorithm, LZ77, LZ78, and gzip. The bzip2 was developed by Seward (77) and used the block sort algorithm giving speedy results.

In order to estimate the complexity of a physiological signal using compression, different approaches have been used, such as an increase/decrease coding system using a binary (30, 78, 79) or ternary alphabet (80, 81).

Compression also has been used for research purposes in a wide variety of fields such as literature (82), music (83), computer virus and internet (84) traffic, but only in 2004, it was first applied in HRV time series by Ferrario et al. (85). Here, compression demonstrated to differentiate healthy fetuses from unhealthy ones. In fact, the former group complexity calculated with LZ achieved similar results to random noise (meaning high complexity), while in the latter group, its complexity was lower, showing sinusoidal patterns. The applications of compression in health research range from event detection [such as epileptic seizure (86), the onset of ventricular tachycardia or fibrillation (87) and changes from sleep to waking state in-depth anesthesia (88)], characterizing neural spike trains (89), fHR biometric identification (90) or in DNA sequences studies (91). A distinct approach to applying compression on a time series uses the normalized compression distance (NCD) measure, a dissimilarity learning approach first used in fHR by Santos et al. (37).

**Figure 4:**
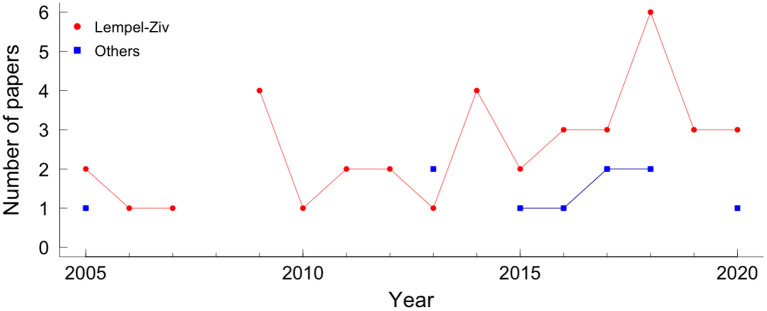
Papers using compression in fetal heart rate, by year. The red circle represents the Lempel-Ziv compressor and the blue square represents the other compressors.

From all 270 papers included in this review, 46 (17%) show results with compression. Its usage throughout recent years can be seen in Figure 4.

### 3.3. Fractal Analysis

Fractality indices quantify self-similarity and fractal- or multifractal-like behaviors. The heart rate fluctuates on different timescales and is similar to itself, which is a good premise for a fractal analysis approach (30).

**Figure 5:**
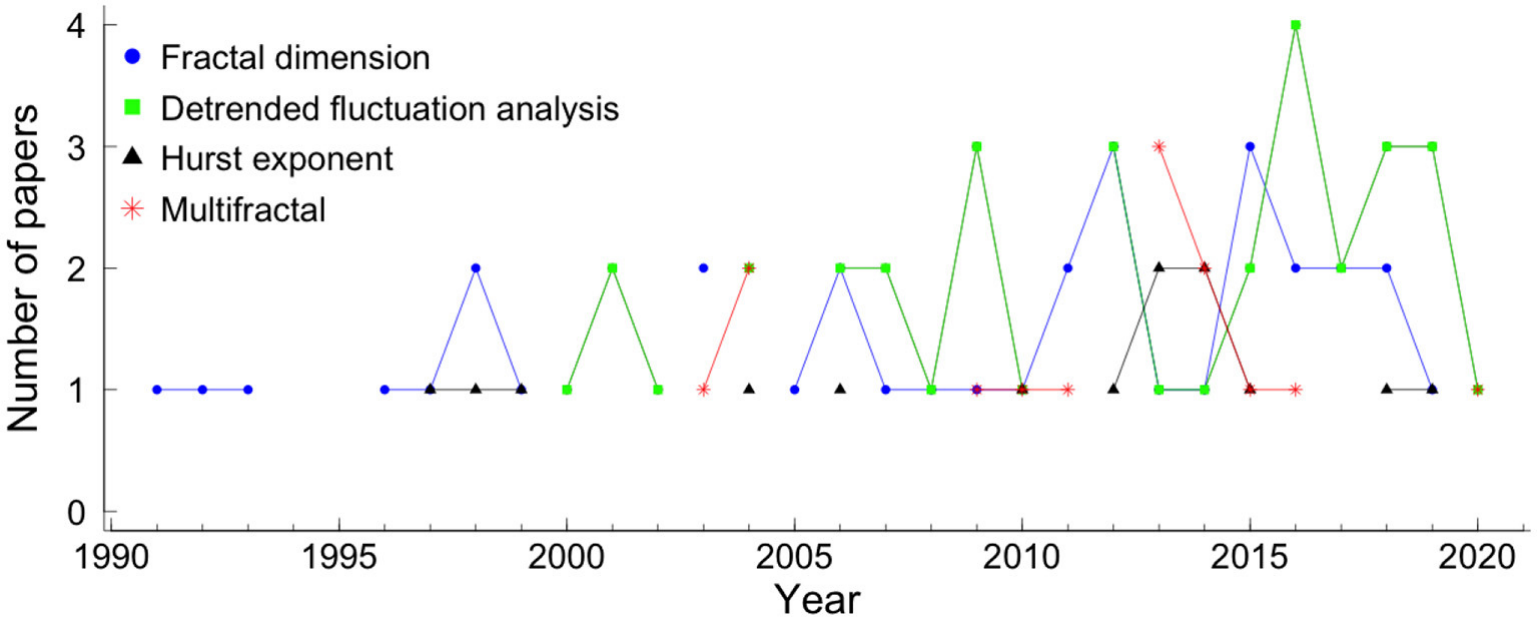
Papers using fractal analysis in fetal heart rate, by year. The blue circle represents the fractal dimension measure, the green square represents the detrended fluctuation analysis, the black triangle represents the Hurst exponent, the red star represents the multifractal analysis.

Of all 270 papers included in this review, 28.1% applied fractal analysis. More specifically, 35 (13.0%) used detrended fluctuation analysis (DFA), 34 (12.6%) show results with fractal dimension (FD), 14 (5.2%) used Hurst exponent and 14 (5.2%) multifractal analysis (see Figure 1). Figure 5 shows the number of papers that applied measures of fractal analysis by year.

Fractal dimension, Hurst exponent, and DFA are described in sections 3.3.1–3.3.3, respectively. The multifractal analysis describes more complex signals than those fully characterized by a monofractal model but requires many local and theoretically infinite exponents to characterize their scaling properties completely. The multifractal detrended fluctuation analysis (MF-DFA), the most applied multifractal method in the papers covered by the systematic review, is described in section 3.3.4.

#### 3.3.1. Fractal Dimension (FD)

A fractal dimension (FD) is a statistical index of how the detail in a pattern changes with the scale at which it is measured. The FD appears to provide a measure of how much space an object occupies between Euclidean dimensions. The higher the FD value, the more irregular the signal is and, therefore, the more self-similar the signal will be.

Of the various algorithms available to calculate the FD of a time series, the four most used are the algorithms proposed by Katz (92) and Higuchi (93, 94), the correlation dimension, and the box-counting dimension (95). More details on the FD calculation algorithms of a time series can found at Henriques et al. (96).

#### 3.3.2. Hurst Exponent

Hurst exponent (HE) or Hurst coefficient is a dimensionless estimator used to evaluate the self-similarity and the long-range correlation properties of time series (97). There are many algorithms to estimate the HE parameter in the literature. The oldest is the so-called rescaled range analysis (R/S) popularized by Mandelbrot and Wallis (98, 99) and it is defined in terms of the asymptotic behavior of the rescaled range (a statistical measure of the variability of a time series). Alternative methods to estimate HE include detrended fluctuation analysis (100, 101), periodogram regression (102), aggregated variances (103), local Whittle's estimator (104), first return method (105), wavelet analysis (106), both in the time domain and frequency domain. Furthermore, there is a relation between HE and the FD, given by *FD* = *E*+1−*HE*, where *E* is the Euclidean dimension, which for time series is 1 obtaining their relationship *FD* = 2−*HE* (107). The *HE* may range between 0 and 1 and can indicate:

- 0 < *HE* < 0.5: time series has long-range anti-correlations;
- *HE* = 0.5: there is no correlation in the time series;
- 0.5 < *HE* < 1: there are long-range correlations in the time series;
- *HE* = 1: the time series is defined self-similar, i.e., it has a perfect correlation between increments.

#### 3.3.3. Detrended Fluctuation Analysis (DFA)

Detrended fluctuation analysis (DFA) quantifies intrinsic fractal-like (short and long-range) correlation properties of dynamic systems (101). Two advantages of DFA over conventional methods (such as the HE method) are that this method allows the detection of intrinsic self-similarity embedded in a non-stationary time series and also avoids the detection of apparent self-similarity (108).

To execute the DFA algorithm the first step is to integrate the time series (of length *N*). The next step is to split the integrated time series into *N*_*n*_ windows of equal length *n*. Then, a least-squares line is fitted to the data, in each window of length *n*. The y-coordinate of the straight-line segments is given the name of *y*_*n*_(*k*). Then, the integrated time series is detrended, *y*_*n*_(*k*), in each window. The root mean square fluctuation of this integrated and detrended series is calculated by the following equation:


(6) $$\begin{array}{r} F\left(n\right)=\sqrt{\frac{1}{N}\overset{N}{\underset{k=1}{\sum }}{\left[y\left(k\right)-y_{n}\left(k\right)\right]}^{2}}. \end{array}$$

This algorithm is repeated for all time scales (box sizes) to characterize the relationship between *F*(*n*), the average fluctuation, and the box size, *n*. Normally, *F*(*n*) increases with the size of the window, according to *F*(*n*) ∝ *n*^α^. The α exponent can be viewed as an indicator of the “roughness” of the original time series: the higher the value of α, the smoother the time series:

- if α ≃ 0.5, the time series represents uncorrelated randomness (white noise);
- if α ≃ 1 (1/f-noise), the time series has long-range correlations and exhibits scale-invariant properties;
- if α ≃ 1.5, the time series represents a random walk (Brownian motion).

Usually, the DFA method involves estimating a short-term fractal scaling exponent, α_1_, and a long-term scaling exponent, α_2_.

#### 3.3.4. Multifractal Detrended Fluctuation Analysis (MF-DFA)

The multifractal DFA (MF-DFA) calculation (109, 110) is similar to the DFA since only two additional steps are taking into consideration. The fitting procedure in the MF-DFA can be linear, quadratic, cubic, or higher-order polynomials (MF-DFAm - the *m*^*th*^ order of the MF-DFA) (101, 111, 112). By comparing the results obtained for different MF-DFA orders, it is possible to estimate the order of the polynomial segment trends in the time series (109, 112). The procedure must be repeated for various *n* time scales, as we are interested in how this *q*-dependent fluctuation function depends on the *n* time scale for different *q* values. The other additional step is a *q* dependent averaging procedure obtaining a generalized (multifractal) scaling exponent *h*(*q*). For *q* = 2, the standard DFA procedure is retrieved.

The main problem with the MF-DFA method is that all the steps are deeply dependent on the user's decisions. The Multiscale multifractal analysis (MMA) (71, 113) is a generalization of the MF-DFA method. The method creates a Hurst surface h(q,s), allowing a broader analysis of the fluctuation properties and more stable results. Also, all multifractal methods, including MMA, require a relatively long time series to analyze.

### 3.4. Wavelets Analysis

The first appearance of the term wavelet was in an annex to Haar thesis' (114). However, it is considered that the wavelet theory was developed in the late 1980s by Mallat (115), Daubechies and Bates (116, 117) to meet the needs for adaptive time-frequency analysis applied to signal processing, mathematics, physics, and engineering. Wavelets are functions that satisfy a series of mathematical parameters and are used in the representation of data or other functions. The term wavelet comes from the fluctuation around the axis, integrating to zero (the areas above the axis and below are the same). Wavelet algorithms process information at different scales (or resolutions). The decomposition of a function using wavelets is known as a transformed wavelet, and it has continuous and discrete variations. Due to the ability to decompose functions in frequency and time domains, wavelet functions are powerful tools for signal processing, widely used in data compression, noise elimination, separation of components in the signal, identification of singularities, and auto-similarity detection.

Let ψ_*s, u*_(*t*), *s*, *u* ∈ ℜ, *s* > 0 be a family of functions defined as translations and re-scales of a single function ψ(*t*) ∈ *L*^2^(ℜ), *L*^2^(ℜ) denotes the space of square-integrable functions on ℜ (118),


(7) $$\begin{array}{r} \psi_{s,u}\left(t\right)=\frac{1}{\sqrt{s}}\psi \left(\frac{t-u}{s}\right) \end{array}$$

where *s* is the scaling parameter and *u* the position parameter. The parameter *u* indicates that the function ψ(*t*) was translated on the *t* axis (translation parameter) by a distance equivalent to *u*. The parameter *s* causes a scale change, increasing (if *s* > 1) or decreasing (if *s* < 1) the wavelet formed by the function. The wavelet is defined as a mother wavelet ψ(*t*) [equivalent to ψ_1, 0_(*t*)], with a family of scale and time daughter wavelets $\psi \left(\frac{t-u}{s}\right)$. Therefore, daughter wavelets constitute a family of curves with a shape identical to that of the mother wavelet, displaced in time and scaled in amplitude. In the time domain, the wavelet transform measures the correlation between the *f*(*t*) signal and the daughter wavelets.

The wavelet ψ_*s, u*_(*t*) has the following basic properties:


(8) $$\begin{array}{r} \int_{-\infty }^{\infty }\psi \left(t\right)dt=0\text{ and}\int_{-\infty }^{\infty }|\psi \left(t\right){|}^{2}dt=1. \end{array}$$

The wavelet ψ_*s, u*_(*t*) has to meet the admissibility condition for the transformation to be invertible (116).

The term $\frac{1}{\sqrt{s}}$ is a normalization factor that ensures that the energy of ψ_*s, u*_(*t*) is independent of *s* and *u*, such that:


(9) $$\begin{array}{r} \int_{-\infty }^{\infty }|\psi_{s,u}\left(t\right){|}^{2}dt=\int_{-\infty }^{\infty }|\psi \left(t\right){|}^{2}dt \end{array}$$

The continuous wavelet transform (CWT) of signal *f*(*t*) is defined as:


(10) $$\begin{array}{r} W_{\psi }f\left(s,u\right)=\langle f\left(t\right),\psi_{s,u}\left(t\right)\rangle =\frac{1}{\sqrt{s}}\int_{-\infty }^{\infty }f\left(t\right)\psi \left(\frac{t-u}{s}\right)dt. \end{array}$$

The CWT coefficients *W*_ψ_*f*(*s, u*) can be obtained by continuously varying the scale parameter *s* and the position parameter *u*. For real discrete signals *f*(*n*), as is the case for the fHR signal, *W*_ψ_*f*(*s, u*) can be calculated according to


(11) $$\begin{array}{r} W_{\psi }f\left(s,u\right)=\frac{1}{\sqrt{s}}\overset{N}{\underset{n=1}{\sum }}f\left(n\right)\psi \left(\frac{t-u}{s}\right). \end{array}$$

If *s* is a continuous variable then *W*_ψ_*f*(*s, u*) is called the continuous wavelet transform. However, if *s* = *a*^*j*^ and $u=n\;*u_{0}\;*a^{j}$ where the integers *j* and *n* control the wavelet dilation and translation respectively; *a* is a specified fixed dilation step parameter set at a value greater than 1, and *u*_0_ is the location parameter which must be greater than zero then *W*_ψ_*f*(*s, u*) = *W*_ψ_*f*(*j, u*) is called the discrete wavelet transform (119). A useful property of the wavelet transform is that it can be viewed as the application of a filter bank (each filter corresponds to one scale) (120). Some authors, such as, Zhao et al. (121) and Papadimitriou et al. (122) apply different scale values, but, in practice, *s* = 2^*j*^ and *u*_0_ = 1 are the most popular scale in fHR analysis (123–125).

**Figure 6:**
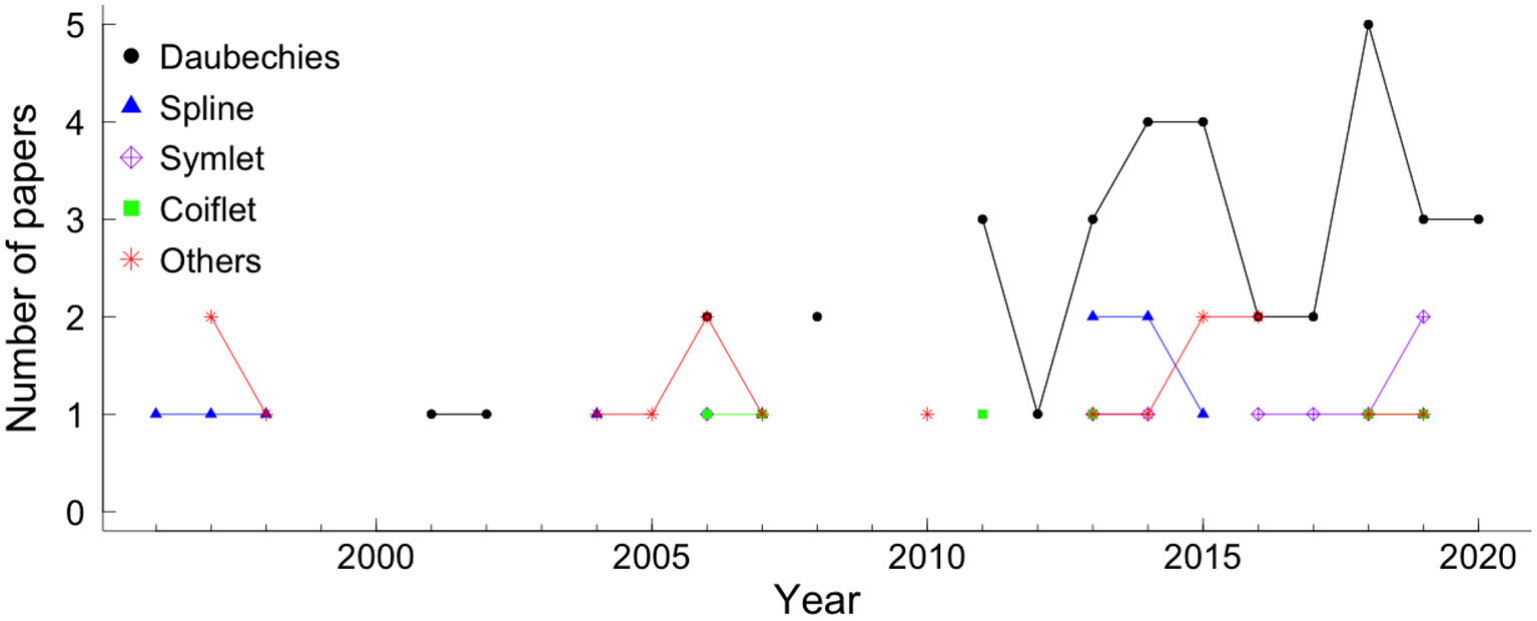
The wavelet families most used in fHR analysis, by year. The colors and symbols represent the wavelet families. The black circle, blue triangle, purple diamond plus, green square, and star red represented Daubechies family, spline family, symlet family, coiflet family, and other families, respectively.

There are a vast number of different mother wavelets, each suitable for different applications. In particular, several wavelet families have been proposed for fHR analysis. From all 270 papers included in this review, 26.3% applied wavelet analysis. The Daubechies (36 papers), spline (11 papers), symlets (11 papers), and coiflet (8 papers) wavelet families were the most applied in fHR analysis (see Figure 1). The application of wavelet analysis in fHR has intensified in the last 10 years, Figure 6.

## 4. Results

There is no doubt of the importance of non-linear measures in fetal monitoring, as they enrich the signal description by providing new indicators for classification and diagnostic purposes. Numerous studies have documented the changes in fHRV during gestation, and fetal growth is associated with a drop in fetal heart rate and increased variability. As non-linear measures started being used, authors started to link their association with different physiological regulatory systems.

**Figure 7:**
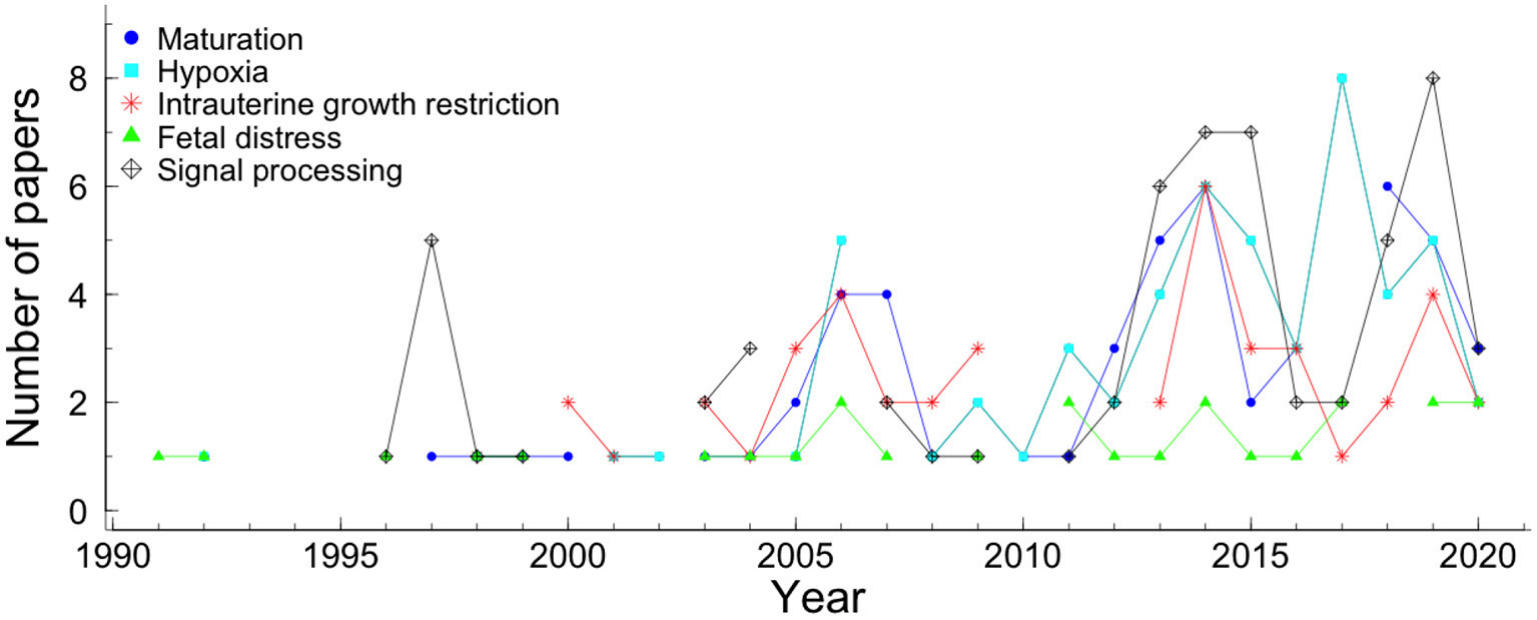
The five most cited research objectives of the papers that applied non-linear methods in fHR, by year of publishing. The colors and symbols represent the different research objectives. The blue circle, cyan square, star red, green triangle, and black diamond plus represent maturation, hypoxia, intrauterine growth restriction, fetal distress, and signal processing, respectively.

The history of non-linear methods reported to fHR summarizes 30 years. However, in the last 15 years, there has been a notable increase in their application to study fHR dynamics (see Figure 7). The main research objectives covered by this systematic review were signal processing (60 papers), hypoxia (56 papers), maturation or gestational age (53 papers), IUGR (44 papers), and fetal well-being or fetal distress (26) (see Figure 7 and Table 2). Also, in Figure 7, the evolution of papers' of the five most cited research objectives is presented per year.

**Table 2:** Research objectives and non-linear methods of the papers selected.

	**Non-linear analysis**													
	**Entropy**					**Comp**		**Fractal**				**Wavelet**	**O**	**T**
**Objectives**	**ApEn**	**SampEn**	**SE**	**MSE**	**O**	**LZ**	**O**	**FD**	**HE**	**DFA**	**O**			
**Healthy**														
Maturation	19	12	4	13	11	7	1	3		12	4	3	14	53
Activity/behavior	10	5	1	2	3	1				3	1	1	3	14
Gender	10	8		2	2	1		1		1			2	11
Presentation	3	2				1		1						3
RM										1				1
Labor	3	2	1	1		1	1			1		1	3	6
Cesarean		1			1			1		1		1	3	4
Preterm	2	2		1	1	1		1		1		1		3
Twins	1	1						1					1	2
Nuchal cord	1													1
FCTE													1	1
Self-organization			1										1	1
Ethnic origins	1													1
**Pathologies**														
Hypoxia	29	28	7	6	11	14	5	11	6	6	7	23	27	56
IUGR	26	13	2	6	3	15		2	1	7	1	2	16	44
Fetal distress	13	7	2	3	4	3	1	4	3	1	2	3	12	26
SIDS	1	1												1
Intrauterine demise				1										1
PPA	1	1						1		1				1
Anencephalus										1			1	1
**Maternal**														
MP	5	1		1		1		1		2	1	2		9
Hypnosis	1	1	1											1
Steroid treatment					1									1
Uterine contraction	2	2	1	1									4	4
**Signals**														
MFCC	1		3		4	1	1						4	7
BI					1		1						1	1
FCEC					1								1	1
Signal Processing	3	7					1	4	2	2		45	28	60
Signal acquisition	1	1												1
fHR baseline										1			4	4
**Others**														
Expert annotation	3	4		1	1	3		4	2	3			4	6
Patterns	5	3						1	1	1			3	9
Fractal value					1			1						1
**Total**	101	82	16	30	38	38	8	34	14	15	15	71	112	

*ApEn, approximate entropy; BI, biometric identification; CD, correlation dimension; Comp, Compressor; DFA, detrended fluctuation analysis; FCEC, fetal cardio-electrohysterographic coupling; FCTE, fetal cardiac timing events; FD, fractal dimension; HE, Hurst exponent; IUGR, intrauterine growth restriction; LZ, Lempel-ziv; MFCC, maternal-fetal cardiac coupling; MSE, multiscale entropy; MP, maternal pathologies; O, others; RM, Respiratory movement; PPA, partial placental abruption; SampEn, sample entropy; SE, Shannon entropy; SIDS, sudden infant death syndrome; T, total*.

Hypoxia can be caused by prolonged or profound asphyxia, an oxygen deficiency due to a pathological change in either fetal or maternal components of the placenta, when there is an exchange of carbon dioxide and oxygen by the fetus during labor. This state leads to an accumulation of carbon dioxide leading to fetal acidemia, resulting in a lower pH in the fetal blood vessels. Early detection of which babies are at risk of acidemia is crucial, as it decreases the chance of a post-diagnosis of cerebral palsy, neonatal encephalopathy, or even death (126). To relate fHR with umbilical artery pH is, therefore, of extreme importance. However, the proper definition of fetal acidemia is still not established as different authors consider different pH cutoffs. Moreover, some authors also include in the definition the value of the base excess or base deficit (127). Some authors defined as “at risk of acidemia” when *pH* < 7.20 (33, 47, 128–136) or *pH* < 7.15 (30, 121, 137–143); others define when *pH* < 7.1 (43, 126, 144–146) or even when *pH* < 7.05 (38, 44, 48, 78, 147–165). Some studies used clinical experts to identify episodes of hypoxia and asphyxia, such as, (54, 166, 167). Another challenge relate to this pathology is to collect enough data for a proper acidemia analysis since prevalence of an acidemic fetus ranges from 0.6 to 3.5% (168, 169). From the 56 papers that aim to analyze of perinatal hypoxia or asphyxia 40 papers applied entropies, 19 applied compression measures, 23 applied fractal measures, and 23 papers applied wavelets analysis.

The development of non-invasive ultrasound techniques allowed a better estimation of gestational age and, therefore, the definition of a crucial fetal outcome: small for gestational age (SGA), which corresponds to fetuses having a weight lower than the 10th percentile adjusted to gestational age. Nevertheless, healthy babies can also be considered SGA, so it is still a challenge to decide whether the small dimensions are due to physiological or pathological conditions (170). Related to SGA fetus, one of the most common pathologies is IUGR. IUGR is a metabolic dysfunction inhibiting the fetus from achieving its average size. With a prevalence of 5−8% in the general population, it can complicate 10–15% of all pregnancies (171). IUGR is the second cause of perinatal mortality, after prematurity (172), and is still an important challenge for diagnosis and management (173). From the 44 papers that aim to study IUGR, 32 papers applied entropies, 15 applied compression measures, 11 fractal and only 2 papers used wavelets.

The effect of an antepartum vs. intrapartum analysis on the complexity indices and the differences in the signal acquisition methods are important to correctly evaluate and assess fetus well-being (174, 175). Throughout pregnancy, the fetus interacts with its environment, as the mother sets the framework for the state and development of the fetus (176). In a study where the mother's breathing was controlled, Van Leeuwen et al. (176) found that the presence or absence of interaction between mother and fetus cardiac activity might be due to maternal respiration. Also, the fetal cardiac system seems to have the capability to adjust its activation rate when responding to external stimuli. Spyridou et al. (177) studied the effect of smoking in fHR and found differences with several linear and non-linear parameters (such as, mutual information, MSE, and compression). In particular, it was shown less complexity for fetus exposed, enhancing its danger. From the 26 papers that aim to study fetal well-being or fetal distress 17 papers applied entropies, 4 applied compression measures, 7 fractal, and only 3 papers used wavelets.

When assessing fHRV, it is essential to control any factor which might confound its interpretation. Some of the most studied factors are the baby's maturation reflected in gestational age, behavioral state, and maternal condition (178). From the 53 papers that aim to study maturation or gestational age 38 papers applied entropies, 7 applied compression measures, 18 fractal and only 3 papers used wavelets. First trimester observations during pregnancy have shown a low intraindividual variation of the fHR, compared to variation between different fetuses (179). Later in pregnancy, Arduini also found this high intraindividual consistency concerning fetal behavioral states' characteristics, particularly fHR, in 2 consecutive days (180). In fact, an association between individual differences in prenatal heart rate and HRV and postnatal neural development has been reported (181). Besides these factors, Gonçalves et al. (131) and Spyridou et al. (182) noted that gender also has an effect on fHR analysis and should be considered, while Tagliaferri et al. (183) found differences on both linear and non-linear indices between different ethnic groups. Gender was also shown to influence maternal heart rate (MHR) (52). Even when twins are considered, sex differences were found both by linear and non-linear indices (184). Fetal presentation at birth has also been studied (185, 186). Reports are stating that breech fetuses have worse neurological outcomes compared to cephalic presentation ones (187, 188). Furthermore, in a study by Choi and Hoh (189), non-linear dynamic indices were able to differentiate normal pregnancies from ones with partial placental abruption with high accuracy, while linear indices were not.

The evaluation of neonatal behavior has shown more success in predicting neurodevelopment disability than neurological examination (190). Therefore, the same approach was adopted for fetal well-being assessment. These fetal behavioral states were introduced back in 1982 in studies combining the assessment of fetal body and eye movements (191). They include calm or non-eye movement sleep state (1F), active or rapid eye movement sleep state (2F), calm wakefulness state (3F), and active wakefulness state (4F). The importance of these definitions in understanding fetal physiology, interpretation of fHR monitoring, and diagnosis of pathological conditions is described with more detail elsewhere (192). There are associations between fetal behavioral states and fHR patterns. 1F is related to a stable baseline with absent or sporadic and short-lasting accelerations; 2F is associated with a stable baseline and frequent accelerations, and it is the most frequent state. 3F is rare and is usually very short in time. It also has a stable baseline but with wide variability and no accelerations. 4F shows repetitive and long-lasting accelerations with eventual returns to the baseline (193).

This field's interest is not only focused on fHR tracings classification. Features like frequency and amplitude traditionally characterize physiological signals. However, these parameters do not provide us with an insight into the regulatory processes underlying the signal dynamics, thus requiring a further extraction of more appropriate features, which has become a difficult task. These difficulties lie in the lack of a priori information on which process belongs to each component (i.e., fetal, maternal, or environmental) and the lack of knowledge on how each component behave (194). Much effort has been put into the signal acquisition and processing models because the extracted features' usability highly relies on the preprocessing steps' quality, such as artifacts removal, interpolation method, segmentation, and detrending signal (30).

In 2013, an open challenge was created, the Physionet Challenge (195), in order to promote the development of advanced signal processing techniques. Many different approaches were suggested, such as wavelet de-denoising, subspace decomposition and reconstruction, adaptive filtering and averaging, matched filtering, and entropy. Most of them followed these four steps: signal processing, maternal heartbeat detection, maternal heartbeat cancelation, fetal heartbeat detection. More information can be found in Di Maria et al. (196). These non-linear methodologies have been studied and applied to retrieve signal with the best quality possible, dismissing as much noise as possible. The preprocessing is even more important when adopting low-cost systems for signal extraction, as is the case of the fetal phonocardiography, which has a poor signal-to-noise ratio (11). From the 60 papers that aim to study signal processing 7 papers applied entropies, 1 applied compression measures, 7 fractal, and 45 papers used wavelets.

Table 2 presents the number of articles that applied each non-linear method for each research objective. Entropy, compression, and fractal measures are most used in classification papers, mainly when applied to analyze the variability of fHR in hypoxia, IUGR, and fetal distress. However, these measures are still underused in studies whose research objective is signal processing. On the other hand, wavelet analysis is most used when the research objective is signal processing (43 papers) or hypoxia (25 papers).

## 5. Discussion

The number of articles probing the use of non-linear measures to assess the fHR signals analysis has been growing in the past decade. Non-linear analysis has been successfully applied in the study of fetal heart rate with several research objectives, such as fetal maturation or gestational age (197–199), fetal gender (182, 200), labor stages (201, 202), cesarean section (51, 203), preterm birth (80, 204), impact of nuchal cord on antenatal (205), fHR baseline (206), behavioral state (207, 208), IUGR (209–213), hypoxia (128, 137, 214, 215), fetal distress (216–218), maternal pathologies (219, 220), and signal processing (198, 221–224). Therefore, it is important that the scientific community is aware of the non-linear methods used depending on the research objective. Additionally, they are not yet used in clinical practice due to some critical concerns that need to be further discussed.

Systems, such as, Omniview SisPorto (225), OxSys (226), NST-Expert, which later became CAFE (227) already automatically deal with CTG assessment. All the fHR processing and analysis in these systems are based on morphological features defined by FIGO guidelines. In some, the CTG is complemented with the ST-analysis method. It has been shown that it slightly improves labor outcomes, but its use is not always possible since it requires an invasive measurement (228). However, none of these systems still integrates non-linear indices, so they can and should be optimized.

When analyzing fHR time series automated, there are two main aspects to contemplate: the signal properties and quality and the clinical characteristics that might influence the measures used. Accordingly, we found that the most studied research objectives in fHR are signal processing, hypoxia, and maturation. Furthermore, the results show that entropy is the most applied measure in fetal heart rate, followed by fractal analysis, wavelet analysis, and the least applied is compression. Although the application of entropy methods stands out, we can see that compression and wavelet analysis methods have been increasingly used in recent years. Also, highlighting the fact that entropy is the oldest method, and that is it has been extensively studied and refined when applied to much different time series (229). On the other hand, wavelets are widely used in signal processing (124, 222) dealing with the signal itself, handling problems such as noise (230) and frequency.

Routinely, the fetal heart rate monitors acquire the beat-to-beat intervals in milliseconds either from Doppler or electrocardiographic signals and then convert them to provide a sequence of instantaneous heart rates in beats per minute (bpm). However, when data is exported, it is sampled, implying an interpolation of signals (132). The sampling rate does not seem to affect many linear parameters, but differences were found when non-linear indices were considered (175). Caution must be taken when defining reference values for irregularity indices, such as entropy, as they depend on the sampling frequency, as shown in (175), where 2 vs. 4 Hz sampling was compared. It is most important not to compare computerized systems for heart rate frequency analysis that use different sampling rates (225, 231).

Several linear methods have been studied as a forecaster of fetal well-being by measuring the interaction between the fetal sympathetic and parasympathetic nervous systems and its effects on fetal cardiovascular activity (232). As the parasympathetic nervous system is more responsible for variations in short-term variability (STV), which usually assesses the beat-to-beat differences, it might be reduced in central nervous system hypoxia/ acidosis. If hypoxia is sustained and increases in severity, it leads to the loss of long-term variability (LTV) (233), resulting in a global decrease of sympathetic and parasympathetic activity. On the other hand, it has been shown that fetal hypoxia's early effects increased short and long-term variability (234). Notwithstanding, many studies verify the weakness of STV and LTV indices in identifying fetal pathologies (235). Furthermore, with fetus maturity throughout pregnancy, an increase in fetal autonomic nervous system activity and the sympathovagal balance is expected. Moreover, motor and neurological delay, as well as damage in specific brain areas with cognitive effects, also affect the STV (236). The IUGR showed a reduction in both components of the autonomic nervous system activity, which modulates heartbeat intervals receiving inputs from the heart, the lungs, and the blood vessels (204, 209, 237).

The indices presented in this review are closely related to fetal heart variability. For instance, in (199) the authors showed that the complexity indices correlate highly with abnormal STV. In (143) the authors also report correlations between the complexity indices ranging from 0.53 to 0.78. Therefore, many studies found a reduction of complexity in the fHR signal associated with hypoxia/ acidosis. However, these indices were not always able to identify fHR from IUGR fetuses (237). Contrastingly, the fractal indices are measures of long-range correlations and long-term memory of time series, therefore, applied mainly in maturation studies.

Furthermore, many of the fetal heart rate analysis methods rely upon stationarity properties like mean, variance, and correlation structure. However, it is known that these fHR properties vary in time through events like uterine contractions. One way to counter this is to select small temporal windows where this property holds. Usually, an interval of 10–20 min is considered the minimum time window to perform the analysis for tracing classification and clinical decision (231, 238). In addition, many of the described measures are parametric measures. The choice of the ideal parameter is far from established in most cases. This heterogeneity limits the possible comparison between the results of different studies. In fact, in various papers, the choice of parameters is neither discussed nor even fully described.

Factors like fetus maturation, behavioral states, and maternal conditions are critical for a good assessment of the fetus and fully understanding their influence in the fHRV is no easy task. Incorporating such variables in predictive models for fetal evaluation will elucidate the importance of individual fHRV and increase its accuracy (178). Maternal psychological conditions such as stress and anxiety influence fHR and maternal hormones transferred via placenta or changes in the oxygen and nutrition supply for the fetus (239, 240). As seen in some results, gender is also a factor that should be taken into consideration. Even when twins are considered, sex differences were found both by linear and non-linear indices (184). Although Park et al. (185) found no significant differences between fetuses with different fetal presentation using spectral and complexity measures such as Lempel-Ziv complexity, ApEn, SampEn, and CD. Gonçalves et al. (186) found differences not only using linear indices but also with non-linear and spectral ones. This example of contrasting results reflects the difficulty and complexity of the fetal assessment. In this case, and according to the authors, the discrepancy might have resulted from different inclusion criteria, conditions for fHR recording, the occurrence of maternal fasting, time interval between acquisition and delivery, and equipment used. Moreover, an interesting study comparing uterine contraction influence on fHRV features between acidemic and non-acidemic fetuses suggested that separating contractions from rest periods improves fHRV analysis in detecting asphyxia during labor (151).

Having as a premise that humans are a result of self-organization and adaptation process and that ontogenetic development reflects phylogenetic development and indices of developmental biology may be helpful in fetal maturation assessment. Many studies addressed here found HRV changes, such as variability increase and pattern formation (204). These universal developmental features deliver appropriate measures of fetal maturation. Therefore, it seems only natural that these self-organization and adaptation features might better understand and identify developmental disorders (241). In fact, attention-deficit hyperactivity disorders in teenage boys were associated with antenatal maternal anxiety (242), which might influence fetal humoral development and autonomic control reproduced in heart rate patterns. This phenomenon, resulting from adverse influences on the fetus explained by epigenetic mechanisms, is called “fetal programming.” Therefore, early identification of fetal developmental disorders is essential as they may not be wholly compensated for later postnatal therapies (243). Many different approaches to fHR processing and analysis have been studied. They range from simple feature extraction methods to more sophisticated classification programs and joining research centers from different countries for joint projects, as the Digi-Newb project (244). Usage of continuous non-invasive evaluation, such as the usage of wearables, have been discussed (27, 245) and will contribute to the patient's care improvement since it will improve data gathering, reducing costs of fetal monitoring. Insurgent approaches are opening new windows on the continuous monitoring of fetal development. A single index cannot retrieve all the information from pathophysiological processes in the fetus's development, so approaches considering both linear and non-linear measures, through multivariate analysis, can improve the assessment of both fetal and maternal well-being.

In (35, 246), time, spectral and complexity indices were used as parameters to discriminate fetuses who were or not in a distressed state. Ferrario et al. (247) conclude that compression quantifies the rate of new patterns arising as the signal evolves, whereas entropy quantifies the recurrence of repetitive patterns. This idea of complementary of different indices is also supported in other papers (130, 145, 167, 247). It seems only logical for such a complex/chaotic system to be evaluated using a multiparameteric approach through advanced classification techniques capable of discriminating fetuses in distress in non-linear regions of a multidimensional space (30). With this approach, Signorini (248) was able to classify IUGR fetuses with accuracy, sensitivity, and specificity above 90%.

Mapping from feature space captured from the fHR signal to the space of decision or diagnosis, many machine learning, and deep learning techniques has been applied. Some examples are: support vector machines (38, 150, 152, 249, 250), conventional methods like k-nearest neighbors (250, 251), a hybrid approach using grammatical evolution (146, 252), artificial neural networks (134), and random forests (49, 253, 254). Cömert and Kocamaz (166) introduced a novel software for comprehensive CTG signals analysis, named CTG Open Access Software (CTG-OAS). This software embeds machine learning tools, such as preprocessing, feature extraction, feature selection, and classification. Fergus et al. (51) demonstrated, using deep learning tools, that machine learning significantly improves the efficiency of detecting cesarean section and vaginal deliveries, compared with the usual visual assessment. In this paper, impressive results were achieved, with both sensitivity and specificity over 90%. One problem of comparing these classification approaches is the apriori definition of the classes. For example, as said before, the definition of acidemia based on the umbilical cord artery's pH varies greatly between studies. Karvelis et al. (255) proposed a classification approach based on weighted voting of clinical annotations. These weights are estimated by using a latent class model with three or four latent classes. Moreover, as these learning techniques depend on the signal and the linear and non-linear measures computed, all the previously referred concerns must be contemplated meticulously. Therefore, the machine and deep learning techniques are particularly resourceful when the measures are thoroughly probed and understood. In this systematic review were found several other articles with machine learning and deep learning techniques. However, the description of these techniques is not the focus of this paper. Future work that analyzes in detail the machine learning and deep learning techniques that apply measures based on fHR dynamics should be considered.

The non-linear methods described in this review are entropy (Shannon, approximate, sample, and multiscale), compression, fractal analysis (fractal dimension, Hurst exponent, detrended fluctuation analysis, and multifractal detrended fluctuation analysis), and wavelet analysis. Other non-linear methods were found in our review, such as Poincaré plot (217), symbolic dynamics (256), phase rectified signal average (210, 211, 257, 258), Lyapunov exponents (259), and recurrence plot analysis (137). In the recent years, Phase Rectified Signal Averaging and derived parameters have been largely applied in fHR analysis to face the problem of accelerations and deceleration which are characteristic of the fHR signal.

Due to the high heterogeneity of study designs, data acquisition methods, aims of the studies, signal processing techniques, and measures (and parameters) used, no meta-analysis was possible to be performed.

This systematic review confirmed the importance of non-linear fetal monitoring measures to analyze the fetus' well-being and pathologies' prediction. The methods probed successfully diagnose pathologies, and new techniques are being proposed and explored to improve that prediction. However, the contradictory results of some of the findings due to the characteristic of the signal, or the sensibility of the measures to some clinical factors, such as fetus sex and gestational age, revealed that the use of these findings in clinical practice is far from reality. These results inhibit the reach for a gold standard or the creation of a decision support system. This review determined the significance of creating several small meta-analyses that might focus on a specific research aim. Additionally, a sizeable multicentric study that can assess the multitude of perspectives involved in the fHR signal analysis is imperative.

## 6. Conclusions

Non-linear measures based on the concepts of chaos, fractality, and complexity gained space in the analysis of fetal heart rate. Good results were achieved in signal processing, in the analysis of fetal well-being, and in diagnosing and predicting pathologies. This systematic review of the non-linear methods (entropy, data compression, fractal analysis, and wavelet analysis) applied to fetal heart rate dynamics includes 270 papers. The application of non-linear methods in the fHR analysis is around 30 years old. However, its application has significantly increased in the last 15 years. This review's main contributions are a detailed description of the non-linear methods most applied in the fHR papers and a discussion of the research objectives. Signal processing, hypoxia, and maturation lead the research objectives of papers that use non-linear analysis in fHR. We found that entropy has been the most used method in classification analysis. Despite, in signal processing, the most used method is wavelet analysis. Machine learning and deep learning techniques should also be analyzed with results in the study of fHR dynamics using linear and non-linear measures. The multitude of conditioning involved in the analysis and classification of the fHR, from the signal characteristics to the effect of some clinical factors in the measures, limits the use of the non-linear measures in clinical practice and difficult the creation of a decision support system. Future studies should focus on a research question and perform a meta-analysis, probing the indices' performance.

## Data Availability Statement

The original contributions presented in the study are included in the article/supplementary material, further inquiries can be directed to the corresponding author/s.

## Author Contributions

JM-S, LA, CC-S, and AT conducted the preliminary literature review by establishing the search method and keywords. MR, JM-S, and AT determined the eligibility, summarized the findings from each study, and compiled them in tables. LC, CC-S, and TH analyzed the disagreements between reviewers. MR, JM-S, LC, AT, and TH writing and editing of the manuscript. CC-S, LA, AT, and TH supervision. All authors revised the paper critically for important intellectual content, made substantial contributions to the conception and design of the article, and agreed to the published version of the manuscript.

## Conflict of Interest

The authors declare that the research was conducted in the absence of any commercial or financial relationships that could be construed as a potential conflict of interest.

## Publisher's Note

All claims expressed in this article are solely those of the authors and do not necessarily represent those of their affiliated organizations, or those of the publisher, the editors and the reviewers. Any product that may be evaluated in this article, or claim that may be made by its manufacturer, is not guaranteed or endorsed by the publisher.
